# Whole blood transcriptome changes following controlled human malaria infection in malaria pre-exposed volunteers correlate with parasite prepatent period

**DOI:** 10.1371/journal.pone.0199392

**Published:** 2018-06-19

**Authors:** Julian Rothen, Carl Murie, Jason Carnes, Atashi Anupama, Salim Abdulla, Mwajuma Chemba, Maxmillian Mpina, Marcel Tanner, B. Kim Lee Sim, Stephen L. Hoffman, Raphael Gottardo, Claudia Daubenberger, Ken Stuart

**Affiliations:** 1 Department of Medical Parasitology and Infection Biology, Swiss Tropical and Public Health Institute, Basel, Switzerland; 2 University of Basel, Basel, Switzerland; 3 Vaccine and Infectious Disease Division, Fred Hutchinson Cancer Research Center, Seattle, Washington, United States of America; 4 Center for Infectious Disease Research, Seattle, Washington, United States of America; 5 Bagamoyo Research and Training Centre, Ifakara Health Institute, Bagamoyo, Tanzania; 6 Sanaria Inc., Rockville, Maryland, United States of America; University College London, UNITED KINGDOM

## Abstract

Malaria continues to be one of mankind’s most devastating diseases despite the many and varied efforts to combat it. Indispensable for malaria elimination and eventual eradication is the development of effective vaccines. Controlled human malaria infection (CHMI) is an invaluable tool for vaccine efficacy assessment and investigation of early immunological and molecular responses against *Plasmodium falciparum* infection. Here, we investigated gene expression changes following CHMI using RNA-Seq. Peripheral blood samples were collected in Bagamoyo, Tanzania, from ten adults who were injected intradermally (ID) with 2.5x10^4^ aseptic, purified, cryopreserved *P*. *falciparum* sporozoites (Sanaria® PfSPZ Challenge). A total of 2,758 genes were identified as differentially expressed following CHMI. Transcriptional changes were most pronounced on day 5 after inoculation, during the clinically silent liver phase. A secondary analysis, grouping the volunteers according to their prepatent period duration, identified 265 genes whose expression levels were linked to time of blood stage parasitemia detection. Gene modules associated with these 265 genes were linked to regulation of transcription, cell cycle, phosphatidylinositol signaling and erythrocyte development. Our study showed that in malaria pre-exposed volunteers, parasite prepatent period in each individual is linked to magnitude and timing of early gene expression changes after ID CHMI.

## Introduction

Malaria caused by *Plasmodium falciparum* continues to be one of mankind’s most devastating infectious diseases despite the many and varied efforts to combat it. It has been eliminated in certain areas of the world by combination of treatment with effective drugs, e.g. chloroquine, and by large scale vector control programs, e.g. through insecticide spraying and insecticide-treated nets, only to resurge as a result of drug and insecticide resistance. In 2016, there was an estimated number of 445,000 deaths related to malaria, the overwhelming majority (90%) occurring in the WHO African Region [1].

An effective malaria vaccine would be a powerful tool for regional elimination and eventual eradication of malaria. Currently the most advanced malaria vaccine candidate is RTS,S/AS01, for which large-scale clinical evaluation in African countries has demonstrated vaccine efficacy against clinical malaria of 34% during the 20 months following dose 1 in children aged 5–17 months [2]. Experimental vaccines comprised of live attenuated *P*. *falciparum* sporozoites have gained increased attention because they are highly effective in providing sterile immunity, i.e. immunity to infection [3–13]. Such vaccines primarily targeting the pre-erythrocytic stage are safe because development of the parasite is arrested before, during or shortly after the liver stage, hence prior to the blood stage during which malaria disease symptoms occur. Several approaches aiming to determine the optimal design and administration mode of such a vaccine are being pursued. Promising results have been obtained in studies using radiation-attenuated sporozoites that were administered by either direct intravenous inoculation [3,5–8] or mosquito bite [12,13], genetically attenuated sporozoites [10,11], or inoculation of volunteers with fully infectious sporozoites under coverage with an anti-malarial drug [4,9]. Besides their application as potential anti-malaria vaccine candidates, aseptic, purified, cryopreserved, whole infectious sporozoites are useful in controlled human malaria infection (CHMI) studies. Targeted infection of volunteers in a controlled environment enables the clear and efficient assessment of vaccine efficacy [14–16], aids the development of anti-malarial drugs [17], and is useful for studying human immune responses to malaria infection [18]. The latter is of particular importance given that we still lack a detailed understanding of the host responses to early stages of *P*. *falciparum* infection.

To overcome aforementioned gaps, high-throughput transcriptome analyses employing microarray and/or RNA-Seq can be valuable. Both technologies have already been used for gene expression profiling of malaria-naïve subjects undergoing anti-malaria vaccination and/or CHMI [19–24], malaria pre-exposed subjects undergoing natural *P*. *falciparum* infection [21,22,25] and the *Plasmodium* parasite itself [25,26]. Collectively, such studies contribute to a more comprehensive understanding of molecular patterns and cell signatures involved in the interaction of the human host with malaria.

Here, we aimed to investigate human transcriptional dynamics during *P*. *falciparum* liver and early asexual blood stage with data from a CHMI study conducted in Bagamoyo, Tanzania in 2014, as described by Shekalage et al. [27]. We investigated the transcriptional responses by RNA-Seq analysis of whole blood from 10 adults from malaria endemic regions following CHMI by intradermal inoculation of PfSPZ Challenge, the first such CHMI ever carried out in malaria pre-exposed adults. Our results add insights into gene pathways and associated molecular functions elicited by the *P*. *falciparum* parasite in malaria-experienced subjects as well as important findings regarding the interplay between differential expression magnitude and malaria asexual parasite prepatent period at an individual level.

## Results

### DE genes shared among subjects after CHMI

**Fig 1:**
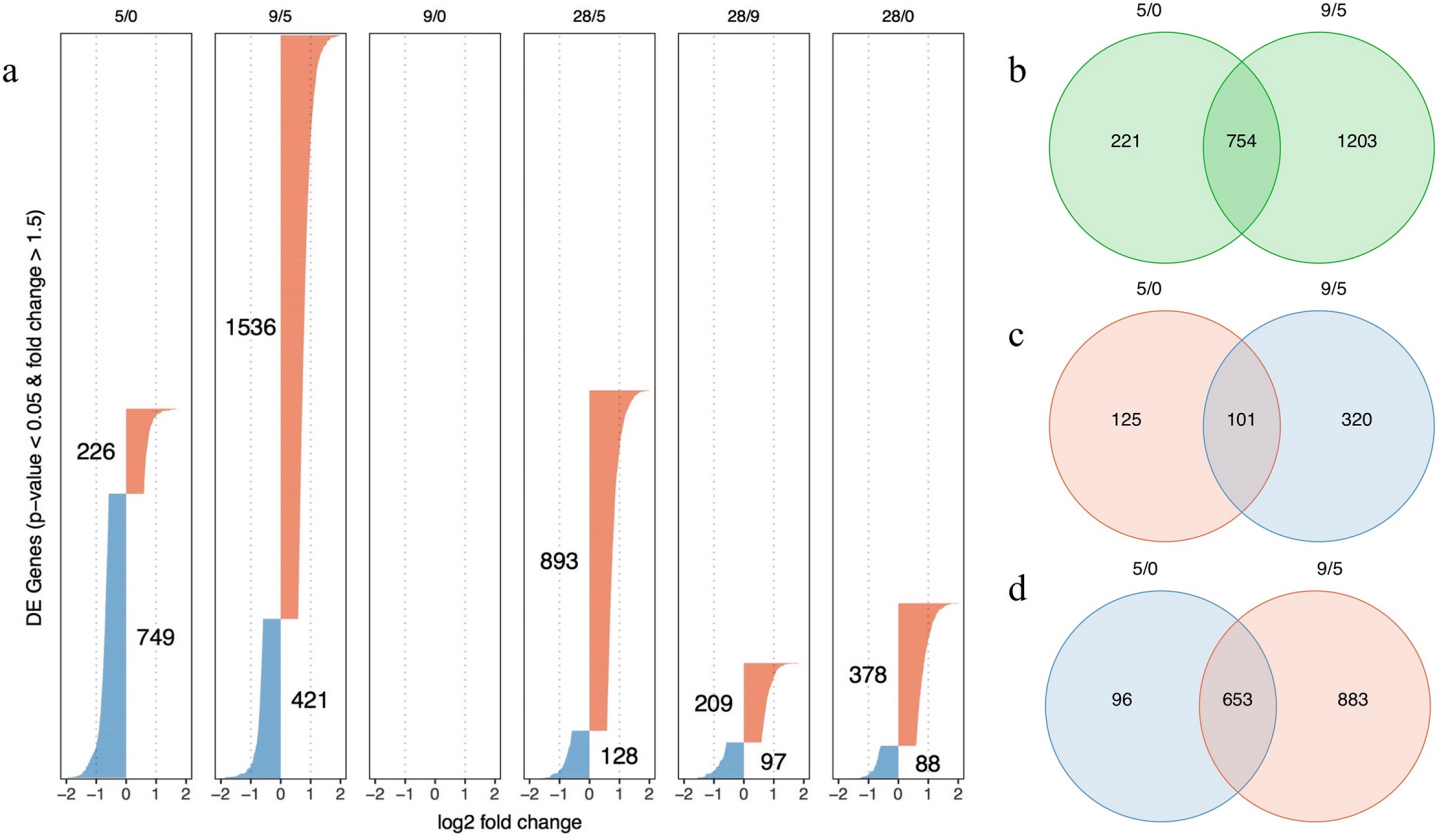
DE genes determined by limma pairwise visit comparison. DE was pronounced at a BH-adjusted p-value < 0.05 and >1.5 fold expression change. (a) DE genes (red: up-regulated genes, blue: down-regulated genes) identified for each tested contrast are visualized as bars. The number of DE genes per contrast is indicated and additionally emphasized by the length of the bars. The bar width / x-axis indicates the log2 fold expression change of each DE gene. The Venn diagrams display the overlaps between (b) all DE genes of contrasts 5/0 and 9/5, (c) the 5/0 up-regulated and 9/5 down-regulated DE genes and (d) the 5/0 down-regulated and 9/5 up-regulated DE genes.

Limma linear modeling was applied to normalized and voom transformed sequence count data to assess temporal gene expression level changes in response to infection with sporozoites. Pairwise comparison of samples collected at baseline, day 5, day 9 and day 28 post CHMI, allowed us to assess the direction and extent of expression changes at the different study visits (Fig 1A). Setting the baseline transcriptional level as a comparator, a multitude of genes were differentially expressed in the blood at day 5 (5/0) and day 28 (28/0) after CHMI. Remarkably, gene expression levels recorded at day 9 post CHMI (9/0) did not differ significantly from the baseline levels. However, extended pairwise comparative analyses revealed substantial numbers of DE genes on day 9 and day 28 relative to day 5 (9/5 and 28/5) and day 28 relative to day 9 (28/9). Most of the DE genes at day 5 (749) were expressed at lower levels in the blood relative to baseline with fewer genes (226) expressed at relatively higher levels. The opposite is true at day 28, when more genes had higher (378) rather than lower levels of expression (88) relative to baseline. The greatest number of DE genes was observed at comparison 9/5 (1,536 genes up, 421 down). Similarly, albeit to a lesser extent, on day 28, 893 genes had increased and 128 genes had decreased expression levels relative to day 5 and 209 genes had increased and 97 had decreased expression levels relative to day 9. Not surprisingly, a significant number of genes were differentially expressed in multiple comparisons. For example, there was a large overlap between the DE genes determined for comparisons 5/0 and 9/5 (Fig 1B). Many of the up-regulated DE genes at 5/0 were down-regulated at 9/5 (Fig 1C) and similarly, the majority of down-regulated DE genes at 5/0 were up-regulated at 9/5 (Fig 1D). Combined, the six pairwise comparisons identified a total of 2,758 unique genes or 16.7% of the total 16,473 genes contained in the data set that were differentially expressed. A list containing the DE genes and their direction of change for each tested contrast is provided in the supplementary section of this manuscript (S1 File).

### GSEA detects DE trends across all genes

Gene set enrichment analysis (GSEA) generated a picture of progression of differential expression over 28 days following CHMI. This analysis incorporated all 16,473 genes in the dataset and ranked the genes in terms of differential expression. GSEA accounts for subtle fold expression changes and simultaneous increased and decreased DE genes in a given gene module [28]. This allowed us to also identify gene dynamics for the 9/0 contrast, despite the absence of DE genes at > 1.5 fold expression changes for this comparison.

**Fig 2:**
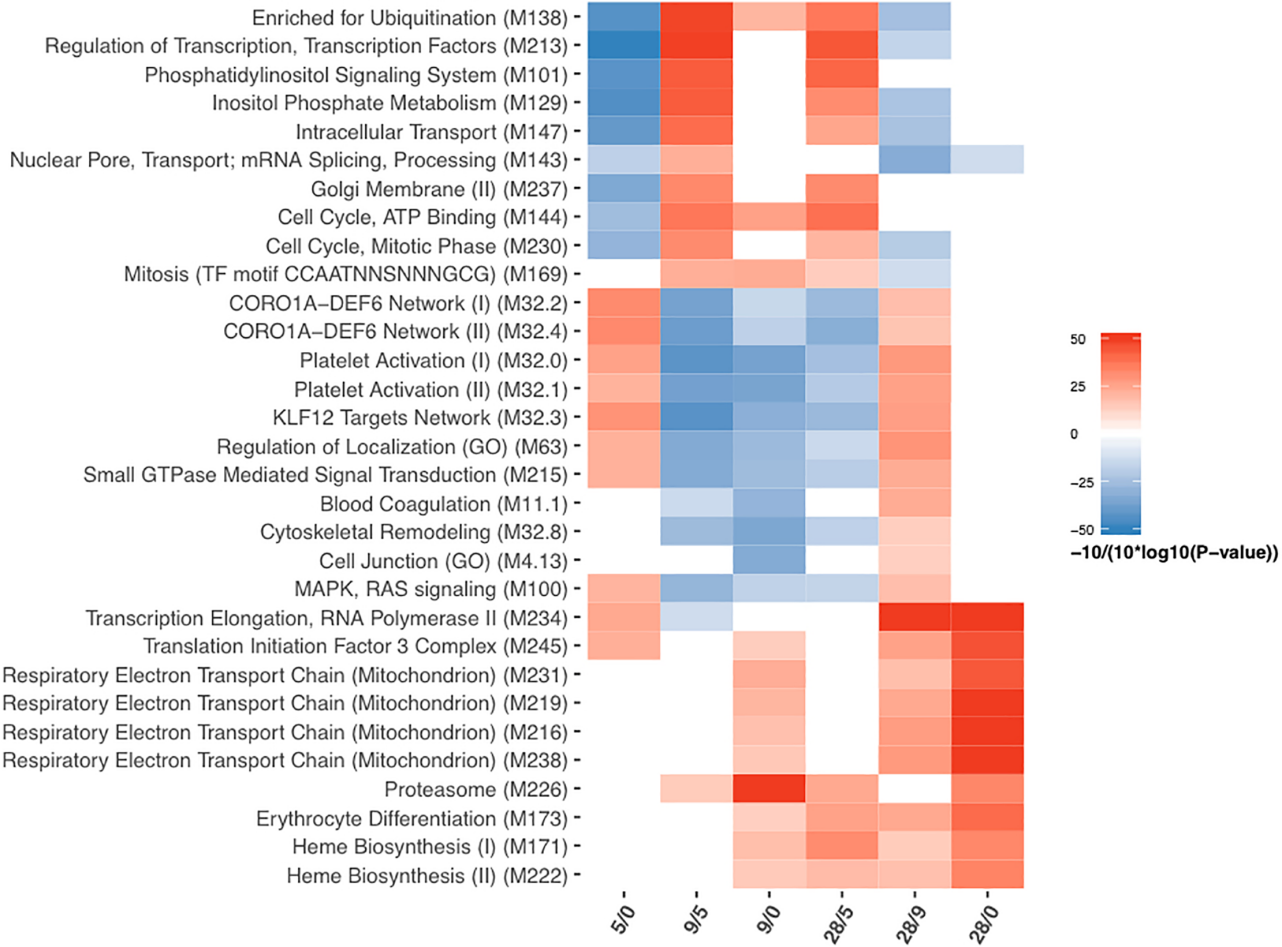
GSEA using camera (limma), visualized as heatmap. Statistical significance is pronounced at a p-value and FDR < 0.05. Red: up-regulated, blue: down-regulated.

GSEA identified several blood transcriptome modules (BTMs) [29] whose expression levels were decreased at comparison 5/0 (Fig 2). These were linked to modules for ubiquitination (M138), transcription factors (M213), and inositol phosphate pathways (M101, M129) as well as cell cycle and intracellular transport (M143, M144, M147, M230, M237). Among BTMs that appeared up-regulated for contrast 5/0 were modules linked to the CORO1A-DEF6 network (M32.2, M32.4), platelet activation (M32.0, M32.1), regulation of localization (M63), signaling events (M100, M215) as well as processes in translation (M245) and transcription (M32.3, M234).

Interestingly, DE modules identified for contrasts 9/5 and 28/5 had a largely reciprocal pattern of expression compared to contrast 5/0. Two BTMs with decreased relative expression at contrast 9/5 that were not detected as DE at contrast 5/0 were associated with blood coagulation (M11.1) and cytoskeletal remodeling (M32.8). The latter BTM was also down-regulated at contrast 28/5. BTMs with no significant change at contrast 5/0 that appeared increased at both contrasts 9/5 and 28/5 were linked to the proteasome (M226) and mitosis transcription factors (M169).

Amongst other BTMs with higher expression levels at contrast 28/5 were modules linked to erythrocyte differentiation and heme biosynthesis (M173, M171, M222). A similar trend in increased expression of red blood cell (RBC) related BTMs was observed for contrasts 9/0, 28/9 and 28/0 as well. Similarly, modules linked to the mitochondrial electron transport chain (M216, M219, M231, M238) and translation and transcriptional processes (M234, M245) were up-regulated with increasing magnitude at contrasts 9/0, 28/9 and 28/0.

Despite not having detected any DE genes for the 9/0 contrast in the first round of our analysis, GSEA revealed a variety of BTMs being differentially expressed at day 9 relative to baseline. In addition to the aforementioned DE modules at contrast 9/0, BTMs linked to ubiquitination (M138), cell cycle (M144), mitosis (M169) and most pronounced, to the proteasome (M226) were identified as up-regulated compared to baseline. The down-regulated modules at 9/0 comparison largely corresponded to the negatively enriched BTMs of the 9/5 and 28/5 comparisons, with the exception of one module linked to cell junction (M4.13). Lastly, contrast 28/9 showed, with exception to the already mentioned modules, DE patterns similar to contrast 5/0. BTMs linked to blood coagulation, cytoskeletal remodeling and cell junction were found to be positively enriched exclusively for the 28/9 contrast.

As a supporting analysis, we repeated the competitive GSEA, using gene sets designed by Chaussabel et al. [30] that incorporate larger numbers of genes per set when compared to the BTMs (S1 Fig). As an additional ancillary analysis, we applied hypergeometric gene set testing, testing for overlaps between the DE genes and BTMs or Chaussabel defined sets (S2 and S3 Figs). For both analyses the identified sets were largely congruent with our initial results using GSEA and BTMs. Additional Chaussabel sets detected were linked to the myeloid lineage and monocyte development (down-regulated at 9/5). In addition, gene sets linked to CD4 cell division and cell cycle (up-regulated at 9/5) and NK cell development and cytotoxicity (down-regulated 5/0) were seen.

### DE gene dynamics and linkage to blood stage parasitemia

**Fig 3:**
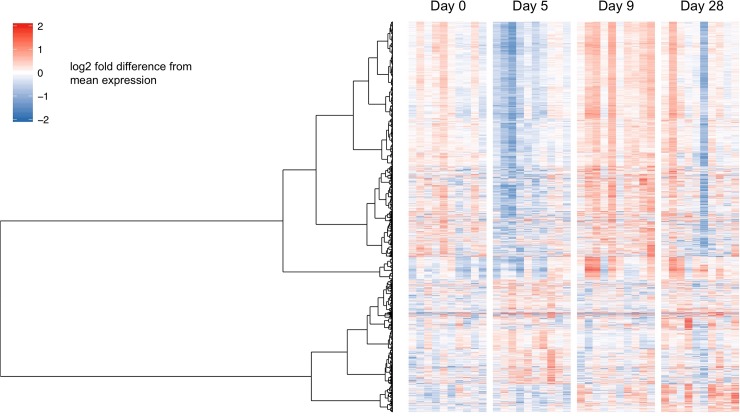
Gene expression patterns in relation to time to detection of blood stage parasitemia. The heatmap displays expression levels of 2,758 DE genes (rows), as determined by limma pairwise comparison of all visits. Subjects (columns) are ordered by increasing pre-patent periods of blood stage parasitemia. Log2 transformed raw counts were centered gene-wise by subtracting the corresponding mean expression values. For visualization purposes, the expression values were limited to 2 and -2. The dendrogram indicates hierarchical clustering of the DE genes based on the Ward method. Two major clusters among the DE genes were identified.

Examining the expression dynamics of the 2,758 DE genes determined in the first part of the study, it became evident that the expression patterns varied not only between different visits but also greatly between volunteers. These expression dynamics are visualized as heatmap in Fig 3, alongside a dendrogram grouping the DE genes in two major clusters. Ordering the heatmap columns based on increasing individual prepatent period, indicated that the majority of DE genes located in the larger cluster seemed to follow a distinct pattern regarding magnitude and direction of expression changes. Primarily at day 5, subjects with a short prepatent period displayed an overall stronger down-regulation of DE genes than subjects with a moderate or long prepatent period. The pronounced down-regulation of genes from the larger cluster at day 28 in one of the subjects was most likely a technical artifact (RIN score of 5.2). This might be a quality issue but nevertheless did not affect the statistical analyses conducted here. We performed both limma linear modeling and competitive GSEA on a reduced set of samples, removing all four samples of two volunteers with the low RIN score samples. Not surprisingly, the number of DE genes determined for the different contrasts were slightly changed, with the ratio between up- and down-regulated DE genes remaining stable. Importantly, this did not influence the GSEA outcome, with identical gene sets being identified as before when analyzing the complete sample set.

Next, we grouped all subjects according to early (9, 9.5, 10), average (11, 11, 11, 11, 11.5, 12) and late (16 days) appearance of blood stage parasitemia measured by qPCR (S4 Fig). Using limma, we performed an F-test to test for differences in temporal expression changes across the three groups. This analysis identified a group of 265 genes linked to parasitemia (S2 File).

**Fig 4:**
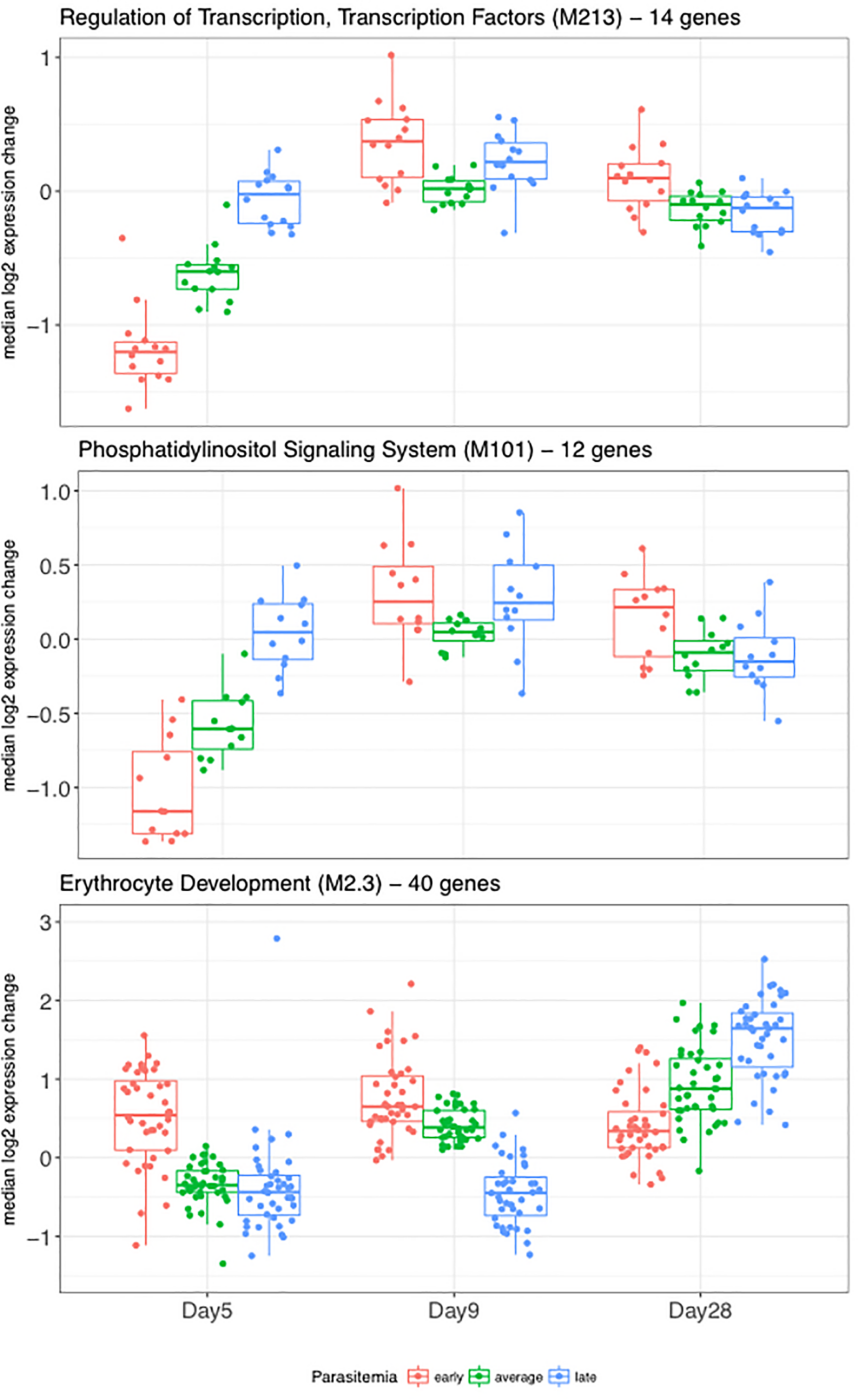
Volunteer gene expression trends visualized as boxplots. Gene expression trends are shown for two differentially expressed BTMs and one gene set linked to parasitemia. Boxplots with gene-wise baseline-subtracted expression values are shown separately for subjects with early (red), average (green) and late (blue) detection of blood stage parasitemia.

**Fig 5:**
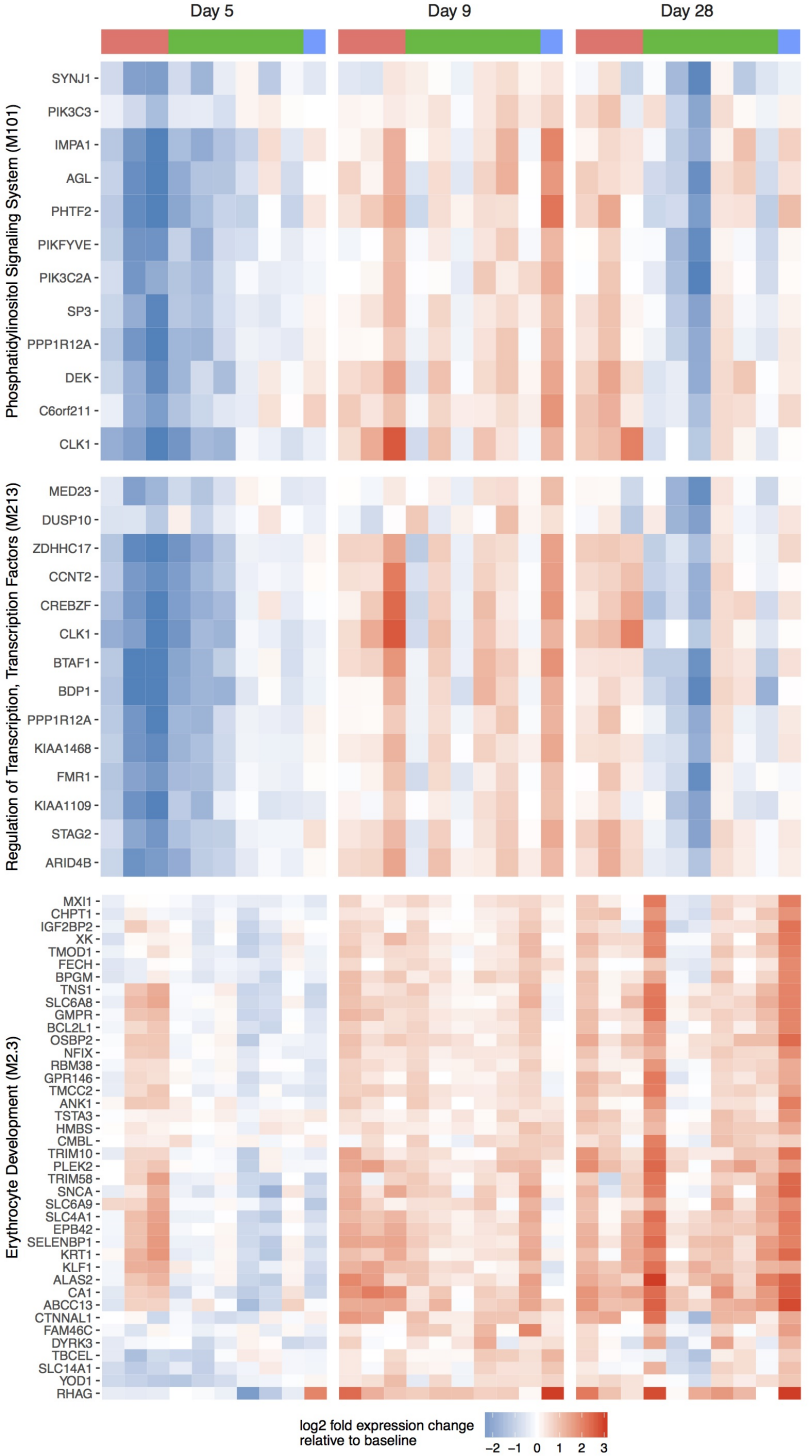
Volunteer gene expression trends visualized as heatmaps. Gene expression trends are shown for two differentially expressed BTMs and one gene set linked to parasitemia. Gene-wise expression levels of the Fig 4 DE modules are visualized as heatmap. Column color bars indicate grouping of subjects into early (red), average (green) and late (blue) group, based on time point of parasitemia detection. Each row corresponds to one gene.

Hypergeometric testing revealed significant overlaps of the 265 DE genes with BTMs linked to regulation of transcription factors (M179, M213), phosphatidylinositol signaling (M101), cell cycle (M144), intracellular transport (M147), ubiquitination (M138) as well as Chaussabel gene sets linked to erythrocyte development (M2.3) and inflammatory processes (M138) (S2 File). Among these BTMs and Chaussabel gene sets, the magnitude of DE gene change was most strongly affected by time to blood stage parasitemia for the 5/0 comparison (Figs 4 and 5). The three subjects (early group) that were within a time window of 4–5 days between day 5 blood collection and parasite detection displayed the strongest down-regulation of genes. Time window differences of 6–7 days (average group; six subjects) or 11 days (one late subject) between day 5 and blood stage parasitemia detection, respectively, correlated with reduced changes to gene expression. Similarly, many genes of the erythrocyte development (M2.3) set displayed increased expression levels in two of the three early subjects already at day 5. By day 9, all other subjects displayed uniform up-regulation of these genes, with the late subject showing the least dynamics.

We assumed that the early and average groups with more robust sample sizes of 3 and 6 volunteers were the main drivers for the here reported results and that the late group with only 1 volunteer had only a weak effect in the statistical model. In order to confirm this, we repeated the limma linear modeling without the late parasitemia subject, comparing only early vs. average subjects. This analysis produced similar results as before, showing an even higher overall number of DE genes (365). Subsequent hypergeometric testing produced the same significant DE gene sets linked to parasitemia. Taken together, this confirms our reported results are not driven by the single late subject but by comparison of the early and average volunteers.

### Gene expression changes in relation to leukocyte population frequencies

In order to rule out that the observed transcriptional dynamics were driven by proportional changes in major cell populations, we also integrated hematology data generated by Shekalage et al. [27] into our analysis. We could indeed observe changes of the leukocyte populations in our study population. This is in line with a recent study by Wolfswinkel et al. that reports changes in total and differential leukocyte counts during the clinically silent liver phase in a controlled human malaria infection in malaria-naïve Dutch volunteers [31]. In our study, a statistically significant increase of lymphocytes, neutrophils and monocytes was observed during the early liver phase of the infection at day 5 (S5 Fig). The increase of neutrophils was even more pronounced at day of parasitemia (e.g. time point of first positive microscopy thick smear). In contrast, lymphocytes numbers were reduced at day of parasitemia. We integrated these WBC dynamics with our limma linear model, investigating whether the magnitude of increase in the leukocyte populations at day 5 correlated with the magnitude of gene expression changes at the same time point. We found no statistical evidence linking the magnitude of cell change to the individual magnitude of gene expression change. WBC changes may certainly influence gene expression patterns but were not the driving force for the subject to subject differences in transcriptional dynamics reported in this study.

## Discussion

We report here for the first time whole blood transcriptome changes over 28 days following intradermal CHMI with aseptic, purified, cryopreserved, infectious PfSPZ in malaria-experienced subjects. Transcriptional changes of hundreds of genes that had increased or decreased relative to their expression levels between days 0 (day of infection), 5, 9 and 28 were identified. Unlike comparable studies that investigated transcriptional responses following vaccination and/or mosquito bite challenge [19–23] this study examined subjects who were infected by intradermal injection with malaria parasites. It also focused on malaria experienced Tanzanian adults who over the course of their life had been repeatedly exposed to *Plasmodium* parasites. It has been shown that such individuals’ immunological responses to CHMI are different from those of malaria-naïve subjects. In Tanzanians, a stronger humoral immune response was recalled after CHMI when compared to Dutch volunteers undergoing identical challenge conditions [32]. Naturally acquired immunity in Kenyans strongly impacts on parasite multiplication rate observed after CHMI, calling for qPCR based molecular monitoring tools in addition to blood slide microscopy for parasite detection [33].

Our study aimed to provide comprehensive insights into early host transcriptional responses occurring during the pre-erythrocytic developmental stage. Since this stage is clinically silent, it is only possible to be studied during a CHMI with parasite strain and infectious doses defined. Therefore, we collected whole blood samples at days 0, 5 and 9 after intradermal CHMI covering this under-researched, early infection period.

A surprising finding of our study was the modest transcriptional changes recorded at day 9 relative to baseline. Although several DE gene sets were later identified by GSEA for this contrast (Fig 2), the absolute expression level changes on gene level remained all below the DEG threshold (1.5 fold change) and were therefore not picked up by the initial limma pairwise analysis. This was unexpected since day 9 is the time point when parasite transition from the liver to the blood occurs in the first volunteers. This finding supports the hypothesis that the timing of whole blood collection as well as the inclusion of early time points (before day 9) during the clinically silent liver stage needs to be targeted in order to optimally capture transcriptional signals. Also noteworthy are the 400 DE genes identified at day 28 post CHMI relative to baseline (Fig 1A). These changes in expression levels cannot be attributed to the effect of CHMI only. We assume that these changes are the combined result of infection and treatment resulting in parasite clearance and development of cellular immune responses.

Importantly, our studies show that significant changes in transcriptional patterns are already observed on day 5—a time point before parasites reach the blood. This is the time period during which an unknown proportion of the injected PfSPZ have infected liver cells and are rapidly developing into thousands of merozoites. High variation (ranging from day 9 to day 16) of parasite prepatent period measured by qPCR strongly indicates that the load of parasites egressing from the liver varied between individuals. Ultimately, this first wave of malaria parasites determines how rapidly asexual blood stage parasites amass to cross microscopy detection threshold (ranging from day 11.5 to day 19) resulting in anti-malaria treatment [34]. The time between malaria infection and microscopic detection, e.g. the prepatent period, has been shown to be associated with degree of malaria pre-exposure. Volunteers in the Tanzanian CHMI-ID trial, including the 10 subjects analyzed in our study, displayed significantly longer prepatent periods than malaria-naïve Dutch volunteers who underwent a similar CHMI study [32]. Pre-existing immunity in the Tanzanian cohort was evident, with more than 50% of the Tanzanian volunteers having a positive *P*. *falciparum* lysate serology at baseline. Similarly, antibody titers for the *P*. *falciparum* antigens CSP, LSA-1, EXP-1, and AMA-1 or preexisting *P*. *falciparum*-specific IFN-γ responses were reported. Importantly, none of these markers for level of pre-existing immunity was associated with the observed differences in prepatency, suggesting that other immunological parameters need to be assessed as well [32].

Based on the prepatent period, we segregated our volunteers into three groups, namely early (n = 3), average (n = 6) and late (n = 1). Interestingly, the length of the prepatent period is reflected by the extent of observed transcriptional changes in peripheral blood. From the time point of infection across all time points, we identified a total of 265 DE genes (S2 File) whose expression level dynamics correlated statistically with time to parasitemia. The majority of these genes were DE around day 5 (overlap with contrast 5/0 DE genes of 88 and 190 with DE genes of contrast 9/5). This was expected since a majority of DE genes and associated BTMs were identified already by the pairwise visit comparisons for the day 5 contrasts.

Notably, our observation of early gene expression changes is in line with a recent study by Kazmin et al. who reported DE genes in response to mosquito bite challenge as early as day 1 and day 5 after infection [20]. Among differentially expressed gene modules correlating with time to asexual blood stage parasitemia detection at contrast 5/0 were two BTMs linked to regulation of transcription and phosphatidylinositol signaling (Figs 4 and 5, S2 File). Genes contained in these modules displayed in unison stronger down-regulation in volunteers with early to average time to blood stage parasitemia. The trends observed in these BTMs are representative of patterns seen in several other modules such as M5.1 (inflammation), M138 (enriched for ubiquitination), M144 (cell cycle, ATP binding) and M147 (intracellular transport) and M179 (enriched for TF motif PAX3). A similar, although reciprocal pattern was observed for genes belonging to gene set M2.3, linked to erythrocyte development (Figs 4 and 5, bottom panel). Genes of this set displayed increased expression levels in two of the three early subjects already at day 5. By day 9, all other subjects displayed uniform up-regulation of these genes, with the late subject showing the least dynamics.

Combined, our observations of individual´s prepatent period interlinked to the magnitude of differential expression on day 5 strongly suggest that blood collection timing is critical and should be conducted at more frequent intervals, additionally covering early time points between days 1 to 4 post CHMI. Capturing time points with the highest transcriptional expression changes might depend on the size of the parasite load multiplying in the liver. Similar studies involving malaria naïve volunteers without pre-existing immunity and with more uniform prepatent periods would shed more light on this hypothesis.

Some of the BTMs identified here as DE have been reported in similar studies that investigated transcriptional responses following controlled infection with *P*. *falciparum* or vaccination. We can only draw limited conclusions when comparing our results with these studies, given the differences in study participants (malaria-naïve or vaccinated vs. pre-exposed subjects), challenge model (mosquito bite vs. intradermal injection) and time point of gene expression assessment. However, there are some interesting parallels to our results: The up-regulation of genes in the proteasome module observed strongest at day 9 and significant at day 28 post CHMI has been reported in response to candidate malaria vaccines TRAP and RTS,S [19,23]. The proteasome is known to play a key function in MHC protein processing and antigen presentation [23], the genes in this module could therefore be of special interest regarding the development of adaptive immune responses against *P*. *falciparum*. The study by Dunachie et al. [19] further reported the antigen processing and presentation pathway and phosphotidylinositol signaling system to be key modules invoked by antigen stimulation after vaccination and the latter to be correlated with time to parasitemia in subsequent challenge by mosquito bite [19]. Interestingly, in our case of intradermal PfSPZ CHMI, we found this pathway to be negatively correlated at day 5. The up-regulation of genes in the MAPK RAS signaling module is an interesting parallel to a finding of Ockenhouse et al., who reported activation of MAP kinases by natural acquired *P*. *falciparum* infection. The same study reported over expression of genes linked to the GO term “protein ubiquitination”following mosquito bite challenge of malaria-naïve subjects. This is an interesting parallel to our BTM linked to ubiquitination that was found up-regulated at 9/0 and down-regulated at 5/0 [21]. Cell cycle related modules have been reported to be affected after RTS,S vaccination and homologous challenge. Interestingly, the same study reported enrichment of genes in NK and monocyte pathways following vaccination and homologous challenge [20].

Studies in malaria mouse models have revealed that liver stage infection results in accumulation of NKT and NK cells in liver tissue and that these cell subsets are involved in parasite protective immune responses [35]. In gene modules defined by the hypergeometric overlap testing, we found that two gene modules (M7.2 and S1) associated with NK cell biology are down-regulated on day 5. These data could indicate that in humans NK cell subsets are recruited from peripheral blood into the liver during the pre-erythrocytic stage infection (S2 Fig).

It should be noted, that the classification of gene and/or module expression change in up- or down-regulated as reported in our study, might not always be the best way of describing the underlying biological or cellular dynamics. By using peripheral blood as starting material for mRNA extraction, abundance or absence of certain transcripts could either reflect general down-regulation of genes within cells or extravasation and recruitment of cells expressing the respective transcripts to other body compartments. For the sake of interpretation it might therefore be sensible to evaluate a module as changed/unchanged rather than focusing on direction of change.

We acknowledge limitations to our study: First, we did not analyze transcriptional dynamics in control subjects uninfected with sporozoites. This could be a minor concern since the samples collected at day 0 served as individual baseline for each subject. Second, the sample size of 10 volunteers limits the generalization of our findings. In our ongoing studies with Tanzanian volunteers undergoing intravenous vaccination and challenge with *P*. *falciparum* sporozoites, we will be able to reconcile our observations in a second, independent cohort of similar origin from Tanzania. This will include a more frequent sample collection and comparison of protected vs. non-protected subjects.

## Conclusion

This study demonstrates that the wide window of parasite prepatent periods in Tanzanian volunteers, most likely due to different levels of pre-existing immunity or natural resistance, is of importance in evaluating transcriptional responses to CHMI. We found that magnitude and timing of early gene expression changes varied greatly among 10 study subjects, coinciding with the individual’s parasite prepatent period. Since optimal sampling time points for each individual are difficult to establish beforehand, we suggest including frequent sampling of blood collections during early stages of infections to capture the short lived transcriptional dynamics of cell populations circulating in the peripheral blood.

## Material and methods

### Ethics statement

All volunteers gave written informed consent before screening and being enrolled in the study. The trial was performed in accordance with Good Clinical Practices, an Investigational New Drug (IND) application filed with the U.S. Food and Drug Administration (US FDA) (IND 14267), and an Investigational Medical Product Dossier (IMPD) filed with the Tanzanian Food and Drug Administration (TFDA). The protocol was approved by institutional review boards (IRBs) of the Ifakara Health Institute (IHI/IRB/No25) and National Institute for Medical Research Tanzania (NIMR/HQ/R.8a/Vol.IX/1217), and the Ethikkommission beider Basel (EKBB), Basel, Switzerland (EKBB 319/11). The protocol was also approved by TFDA (Ref. No. CE.57/180/04A/50), and the trial was registered at ClinicalTrials.gov (registration ID: NCT01540903, date of registration: 23/02/2012).

### Clinical trial design and sample collection

Details of volunteers enrolled and study procedure are given in Shekalaghe et al., 2014 [27]. The single center, double-blind, randomized, controlled trial was conducted in Bagamoyo, Tanzania between February and August 2012. Briefly, 30 healthy male volunteers 20 to 35 years of age were recruited from institutions of higher learning in Dar es Salaam. Screening for eligibility took place at the Clinical Trial Unit of the Ifakara Health Institute in Bagamoyo. Volunteers were screened using predetermined inclusion and exclusion criteria based on clinical examinations and laboratory tests. Tests included medical history and physical examinations, standard hematology, biochemistry and test for malaria, human immunodeficiency virus, hepatitis B and C, and sickle cell disease. Volunteers were injected intradermally with 10,000 (*N* = 12) or 25,000 (*N* = 12) aseptic, purified, cryopreserved *P*. *falciparum* sporozoites or normal saline (*N* = 6). From day 5 after the controlled human malaria infection (CHMI), thick blood smears were obtained regularly to detect blood parasitemia. Volunteers who became microscopy smear positive, were treated with a standard 3-day regimen of arthemether/lumefantrine (Coartem). qPCR analysis for sensitive detection of blood stage parasitemia was carried out retrospectively after volunteers had been diagnosed and treated. The CHMI proved to be safe for all subjects, showing a high infectivity with 11/12 of the low dose and 10/11 of the high dose subjects developing blood parasitemia [27]. Samples for RNA-Seq were collected from the 10 subjects of the high dose (25,000 PfSPZ) group who developed blood stage parasitemia after CHMI. 2.5 ml whole blood was collected into PAXgene tubes on days 0, 5, 9 and 28 of the study, transported to the Bagamoyo research and training centre (BRTC) laboratory and stored at -80°C.

### RNA isolation and sequencing

Poly(A)^+^ RNA was prepared from whole blood in PAXgene Blood RNA tubes that had been stored at -80°C. Following the manufacturer’s protocols, RNA was extracted using the PAXgene Blood Kit (PreAnalytiX) and quantified by spectrophotometry. A total of 1.2 μg of total RNA per sample was processed using the GLOBINclear Human kit (Ambion) in order to remove globin mRNA. The quantity and quality of the RNA was analyzed on a Bioanalyzer Eukaryote Total RNA Nano chip. The average RNA Integrity Number (RIN) score across all 40 samples was 8. Two samples collected at day 28 post CHMI (6.4 and 5.2) were below the recommended minimum RIN threshold of 7. RNAs of all samples were submitted for library preparation and sequencing (Expression Analysis Inc., NC). Sequencing libraries were prepared using the TruSeq Stranded mRNA Library Prep Kit (Illumina), 50 nt paired-end sequence reads were obtained using an Illumina HiSeq 2000 platform and captured as raw sequence data (FASTQ files). All samples were assessed for a sufficient total read count and subsequently passed quality test using FASTQC.

### Data processing and statistical analysis

The reads were aligned with STAR [36] against the UCSC hg38 human reference genome and annotated with RSEM [37] (S6 and S7 Figs), applying the default parameters. Read libraries were normalized with TMM (edgeR) [38] and transformed with voom (limma) [39,40]. Following common practice [41], a total of 5,530 genes exhibiting low counts (< 0.5 counts per million) across all libraries were removed, ultimately leaving 16,473 unique genes in the dataset. For the linear modeling of differential gene expression, we performed three analyses: (1) A linear model with a moderated Bayesian variance estimator was applied to the comparisons between time points. The correlation due to repeated measures across time points for the same subjects was controlled by using subject as a blocking variable in the linear model. The analysis used as one group the 10 high dose subjects who developed blood stage parasitemia and identified the differentially expressed (DE) genes in response to CHMI across time. DE genes were identified with comparisons between the pairwise time points of interest (day 5 vs. day 0 (5/0), 9/5, 9/0, 28/5, 28/9 and 28/0). (2) A secondary analysis was conducted where time to detected asexual blood stage parasitemia was added as a categorical variable (early, average, late) along with an interaction effect (~ parasitemia * day) to the limma linear model. DE genes were identified with an ANOVA-like comparison of all interaction effects using an F-statistics. The null hypothesis being that all interaction effects are zero, and thus time of parasitemia does not have any effect on gene expression changes over time. (3) To determine if leukocyte population frequencies had an impact on differential gene expression, we added the cell counts (reported in S5 Fig) as a continuous covariate along with an interaction effect to the limma linear model (~ day * cell_count). DE genes were identified as in the parasitemia model with an ANOVA-like comparison. The linear modeling was carried out separately for each of the 4 investigated cell populations. For all three analyses, a statistical cutoff of the Benjamini-Hochberg (BH) adjusted p-value less than 0.05 and a minimum 1.5 fold change was used to select DE genes. After the linear modeling, competitive GSEA (camera) [42] was conducted with the blood transcriptome modules (BTM) established by Li et al. [29]. Gene sets described by Chaussabel et al. [30] were used in a confirmatory competitive GSEA analysis. Hypergeometric gene testing (GeneOverlap R package) [43] was performed as an ancillary analysis to support the camera competitive GSEA findings. Given two sets of gene lists (e.g. DE genes at different contrasts and BTMs), this package calculates the overlaps between all pairs of lists from the two sets. Fisher’s exact test is then used to determine the p-value and odds ratio in comparison to a genomic background (the genome size) A statistical cutoff of the BH adjusted p-value less than 0.05 was used for selecting significant modules.

## Supporting information

S1 File DE genes determined by limma pairwise visit comparison. logFC: estimate of the log2-fold-change in gene expression corresponding to the tested contrast; AveExpr: average log2 gene expression level over all visits; t: moderated t-statistic; P-value: raw p-value; adj. p-value: adjusted p-value or q value; Trend: direction of gene expression change.
(XLSX)

S2 File DE genes and gene sets linked to parasitemia. BTMs and gene sets sharing significant overlap with 265 DE genes linked to parasite prepatent period, as determined by hypergeometric testing. FDR: false discovery rate; F: moderated F-statistics.
(XLSX)

S1 Fig GSEA incorporating Chaussabel gene sets. Statistical significance is pronounced at a p-value & FDR < 0.05. Red: up-regulated, blue: down-regulated.
(TIF)

S2 Fig BTM hypergeometric testing. Blood transcriptome modules (BTM) sharing significant overlap with DE genes as determined by hypergeometric overlap testing. Only significant overlaps (BH adj. p-value < 0.05) are shown.
(TIF)

S3 Fig Chaussabel hypergeometric testing. DE Chaussabel gene sets determined by hypergeometric gene set testing. Statistical significance is pronounced at a p-value & FDR < 0.05. Each tile is labeled with the overlap size vs. overall module size.
(TIF)

S4 Fig Volunteer parasitemia data measured by qPCR. Development of asexual blood parasitemia in 10 volunteers as reported 2014 by Shekalaghe et al. [27]. (a) PMR: parasite multiplication rate, determined applying a linear model as described by Douglas et al. [34]. (b) Development of blood parasitemia visualized as line graph. Colored bars (a) and lines (b) indicate grouping of volunteers into early (red), average (green) and late (blue) for RNA-Seq statistical analysis.
(TIF)

S5 Fig Changes of leukocyte population frequencies following CHMI. Boxplots are shown for total leukocytes (a), lymphocytes (b), neutrophils (c) and monocytes (d). Individual volunteers are colored according to detection time point of blood stage parasitemia as early (red), average (green) or late (blue). Bars with asterisk indicate statistically significant changes between visits as determined by paired t-test (*: p-value < 0.05, **: p-value < 0.01, ***: p-value < 0.0001).
(TIF)

S6 Fig Read mapping information. Read mapping to UCSC hg38 reference genome using STAR. Illumina sequencing yielded 58.56 to 82.54 million paired-end reads (mean 69.29 million). STAR successfully mapped an average of 87.13% (63.4% - 94.1%) reads to the human reference genome. Among these reads, 13.25% (11.31–17.5%) mapped to multiple loci (light green), with the remaining reads mapping to unique sequence stretches on the reference genome (dark green). Unmapped reads were mostly too short (97.47%, salmon) indicating impaired sequencing quality. A small fraction of unmapped reads (2.0%) were mapped to too many loci or not mapped to the reference for other reasons (0.53%, red).
(TIF)

S7 Fig Gene count information. Distribution of log2 gene counts after RSEM read quantification. On average, 25.48 million counts were shared across 18,463 (17,513–18,877) gene symbols per sample. Half of these genes exhibit between ~30 to ~1'000 counts. Each 25% of the genes have counts below ~30 or above ~1'000 (up to 1.8 million counts per gene). Across all samples, 22,003 unique genes were covered. Samples are ordered by study day of collection (0,5,9,28) and grouped by subject. Outlier values (> Q3 + 1.5xIQR) are displayed as dots.
(TIF)
