# Supplementary figures and images for: Whole blood transcriptome changes following controlled human malaria infection in malaria pre-exposed volunteers correlate with parasite prepatent period
Source: PLoS One. 2018 Jun 19;13(6):e0199392. doi: 10.1371/journal.pone.0199392 (PMC6007927; doi:10.1371/journal.pone.0199392)

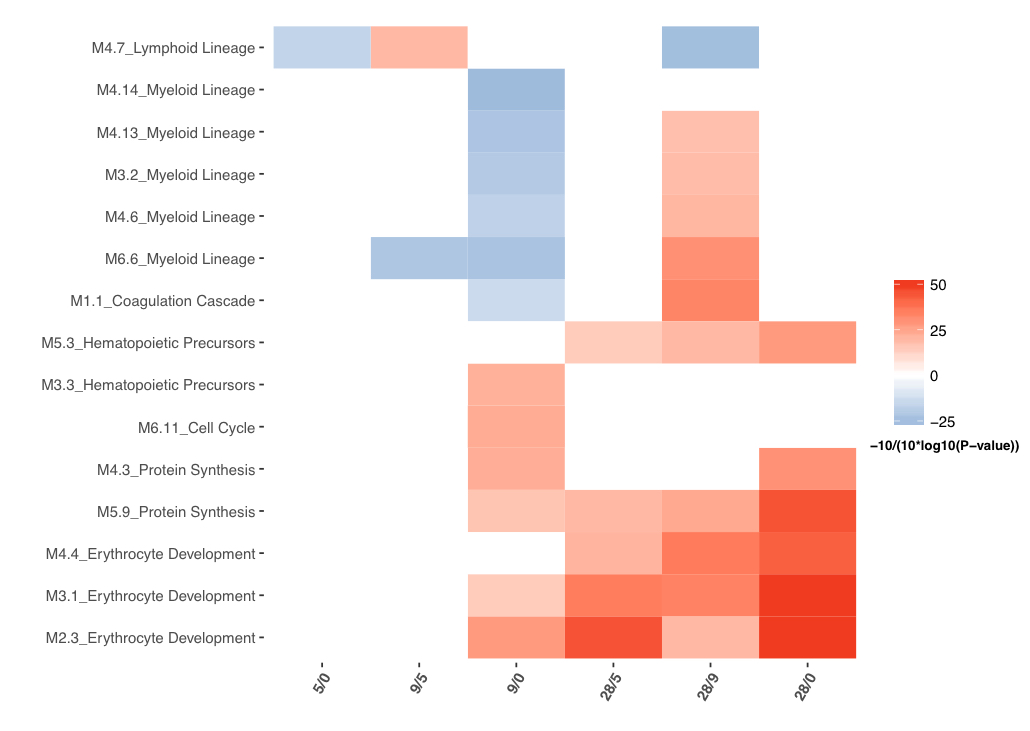

Supplement: S1 Fig — Statistical significance is pronounced at a p-value & FDR < 0.05. Red: up-regulated, blue: down-regulated. (TIF) [file pone.0199392.s003.tif]

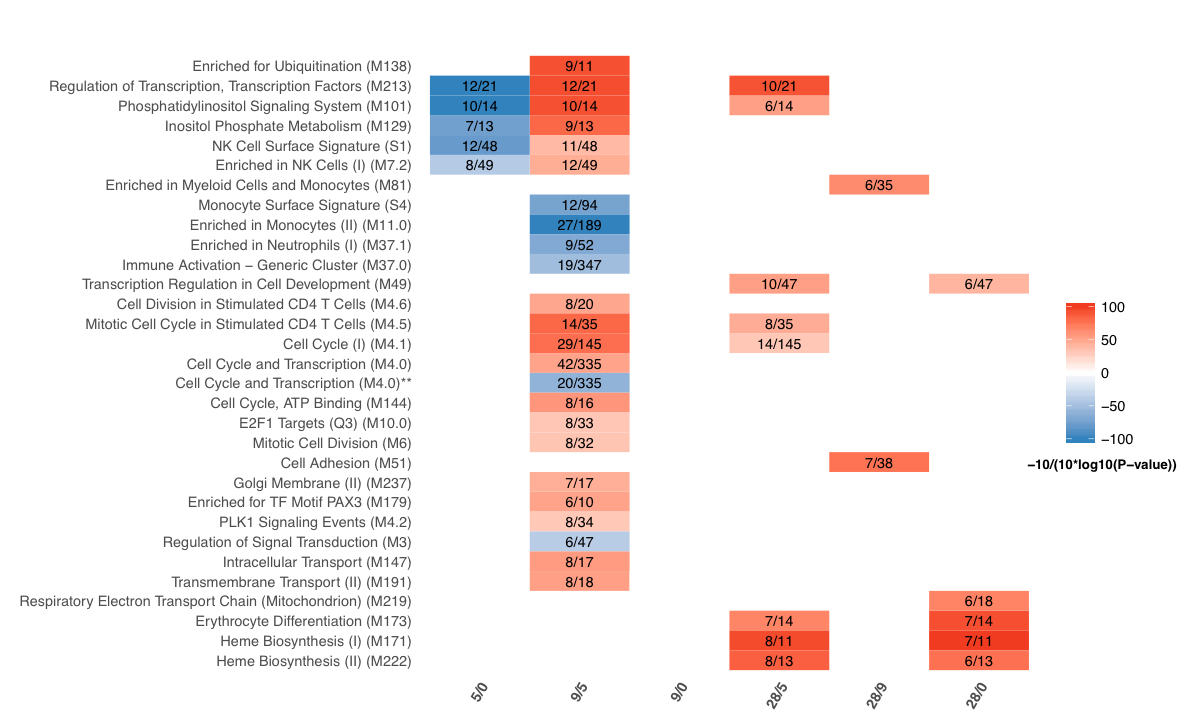

Supplement: S2 Fig — Blood transcriptome modules (BTM) sharing significant overlap with DE genes as determined by hypergeometric overlap testing. Only significant overlaps (BH adj. p-value < 0.05) are shown. (TIF) [file pone.0199392.s004.tif]

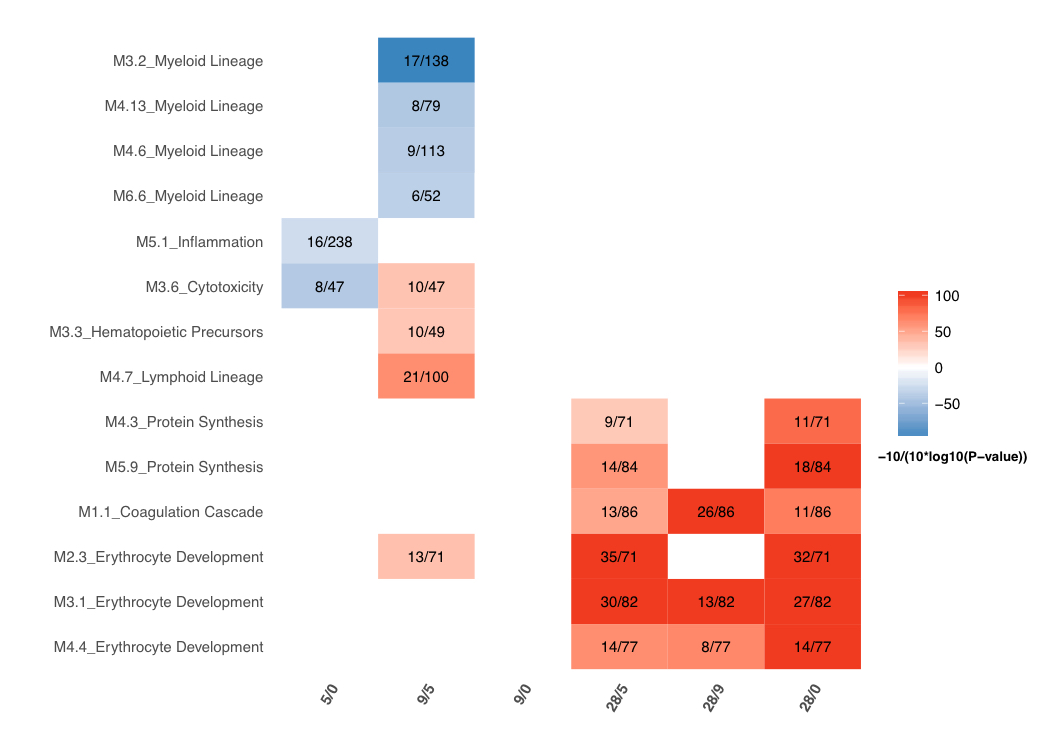

Supplement: S3 Fig — DE Chaussabel gene sets determined by hypergeometric gene set testing. Statistical significance is pronounced at a p-value & FDR < 0.05. Each tile is labeled with the overlap size vs. overall module size. (TIF) [file pone.0199392.s005.tif]

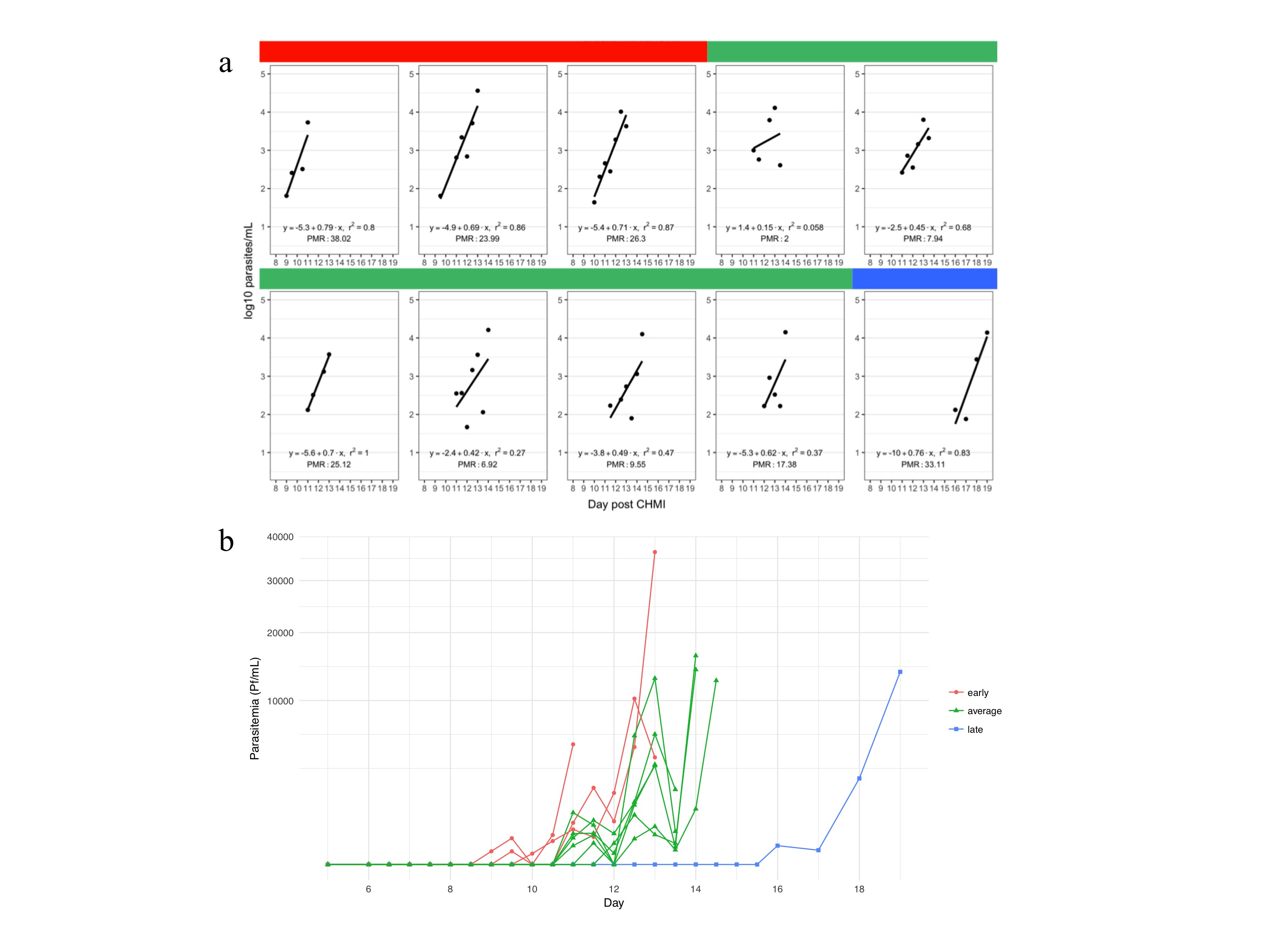

Supplement: S4 Fig — Development of asexual blood parasitemia in 10 volunteers as reported 2014 by Shekalaghe et al. [27]. (a) PMR: parasite multiplication rate, determined applying a linear model as described by Douglas et al. [34]. (b) Development of blood parasitemia visualized as line graph. Colored bars (a) and lines (b) indicate grouping of volunteers into early (red), average (green) and late (blue) for RNA-Seq statistical analysis. (TIF) [file pone.0199392.s006.tif]

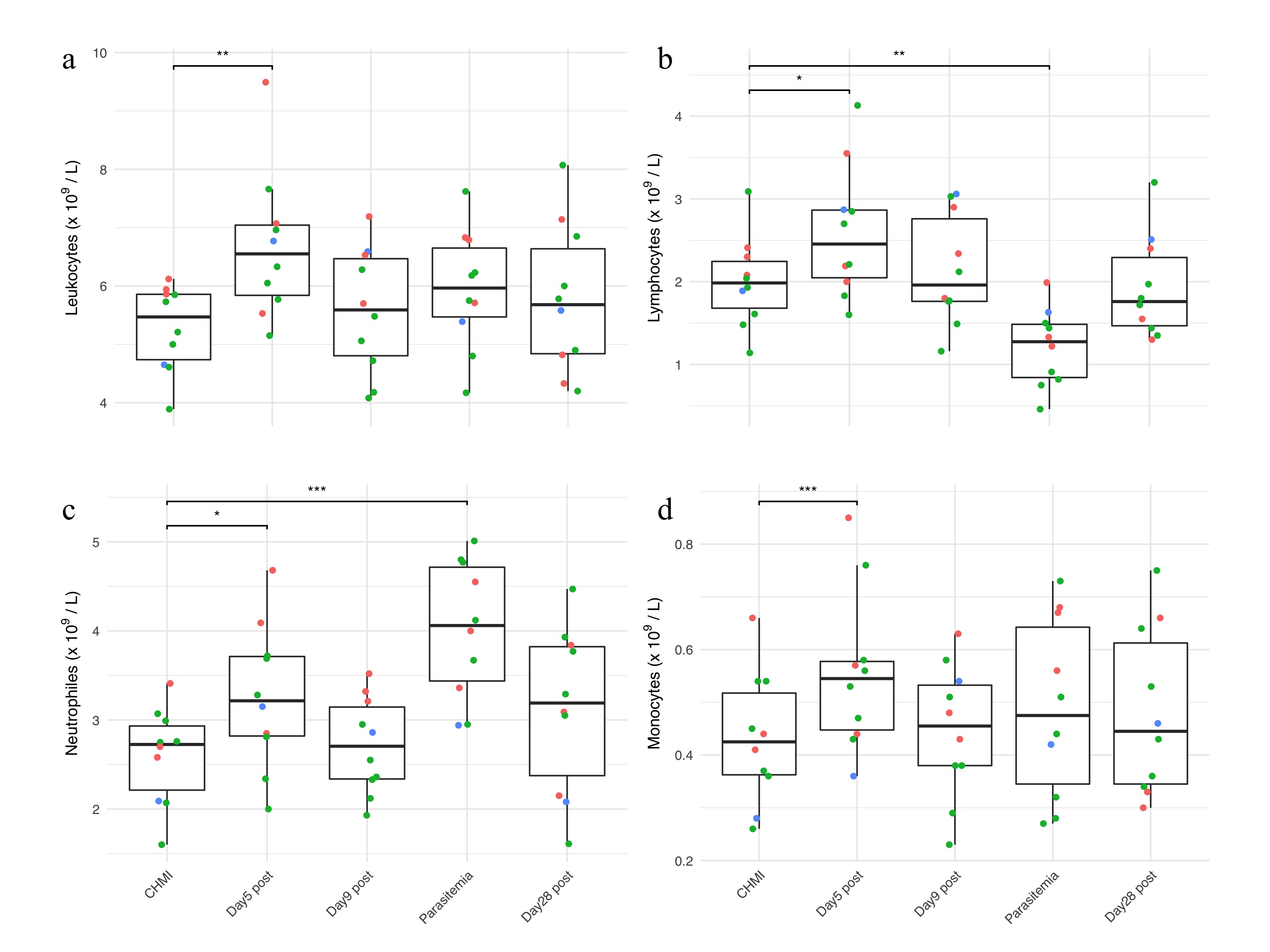

Supplement: S5 Fig — Boxplots are shown for total leukocytes (a), lymphocytes (b), neutrophils (c) and monocytes (d). Individual volunteers are colored according to detection time point of blood stage parasitemia as early (red), average (green) or late (blue). Bars with asterisk indicate statistically significant changes between visits as determined by paired t-test (*: p-value < 0.05, **: p-value < 0.01, ***: p-value < 0.0001). (TIF) [file pone.0199392.s007.tif]

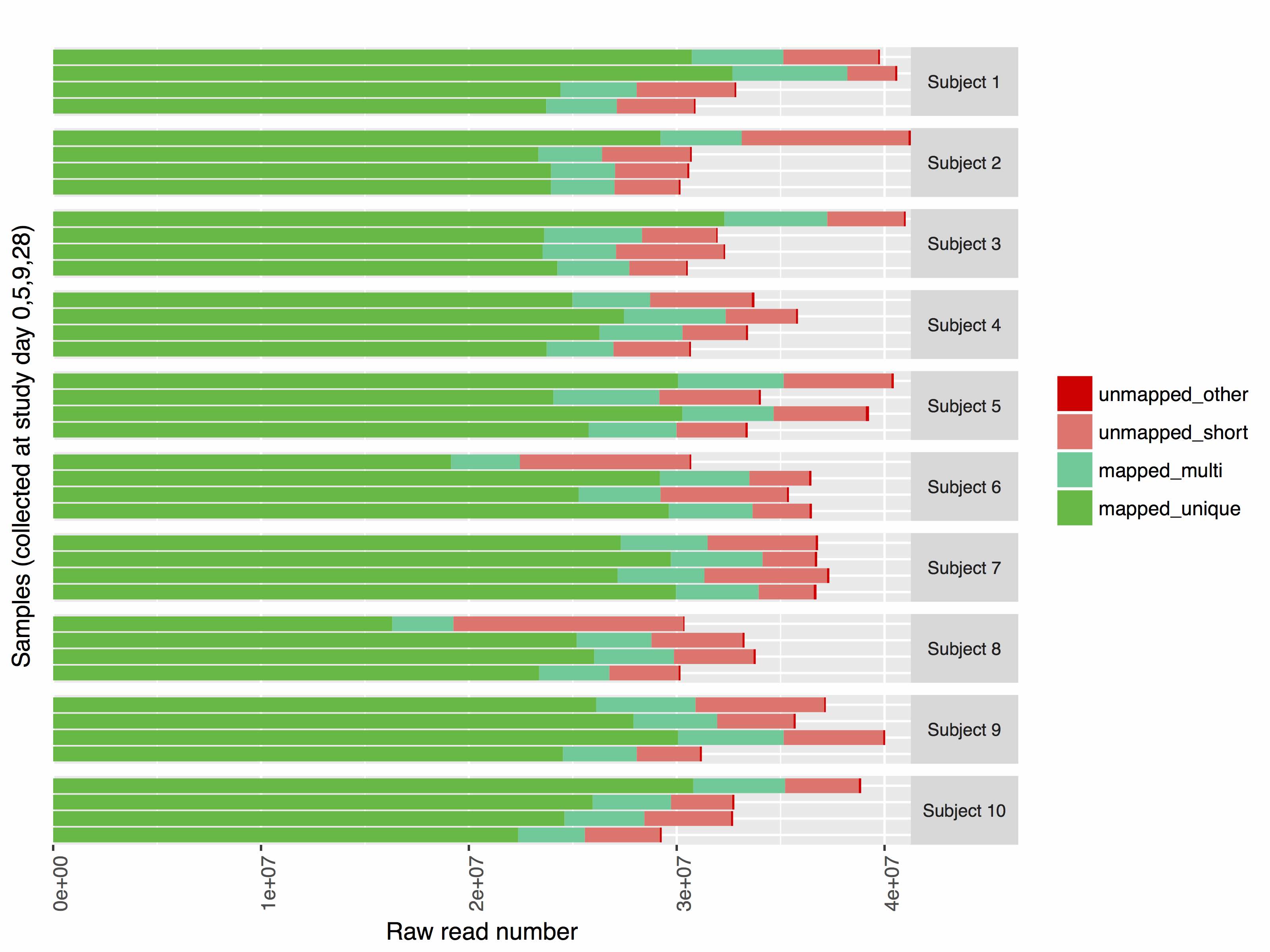

Supplement: S6 Fig — Read mapping to UCSC hg38 reference genome using STAR. Illumina sequencing yielded 58.56 to 82.54 million paired-end reads (mean 69.29 million). STAR successfully mapped an average of 87.13% (63.4% - 94.1%) reads to the human reference genome. Among these reads, 13.25% (11.31–17.5%) mapped to multiple loci (light green), with the remaining reads mapping to unique sequence stretches on the reference genome (dark green). Unmapped reads were mostly too short (97.47%, salmon) indicating impaired sequencing quality. A small fraction of unmapped reads (2.0%) were mapped to too many loci or not mapped to the reference for other reasons (0.53%, red). (TIF) [file pone.0199392.s008.tif]

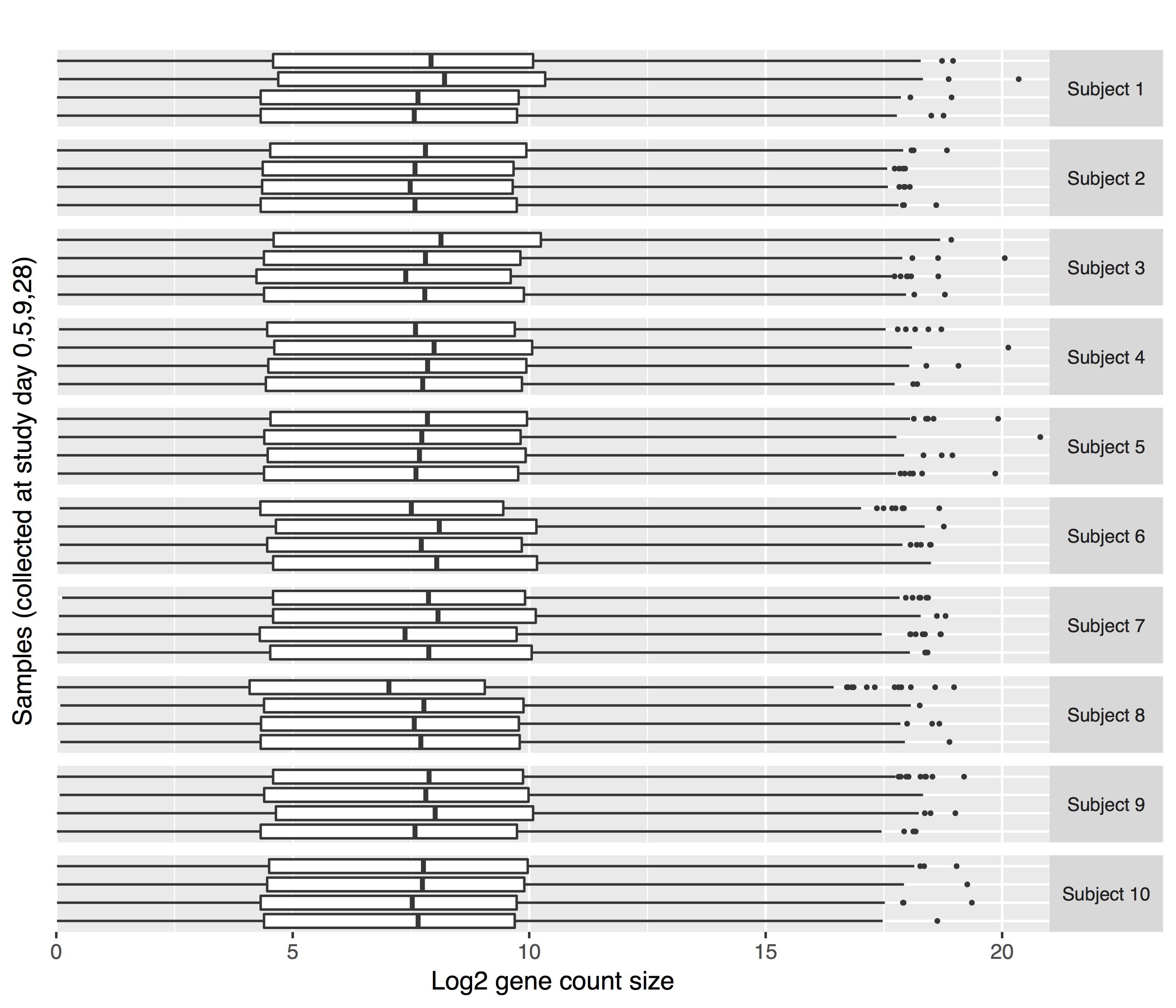

Supplement: S7 Fig — Distribution of log2 gene counts after RSEM read quantification. On average, 25.48 million counts were shared across 18,463 (17,513–18,877) gene symbols per sample. Half of these genes exhibit between ~30 to ~1'000 counts. Each 25% of the genes have counts below ~30 or above ~1'000 (up to 1.8 million counts per gene). Across all samples, 22,003 unique genes were covered. Samples are ordered by study day of collection (0,5,9,28) and grouped by subject. Outlier values (> Q3 + 1.5xIQR) are displayed as dots. (TIF) [file pone.0199392.s009.tif]
